# Interactions between the lung microbiome and host immunity in chronic obstructive pulmonary disease

**DOI:** 10.1002/cdt3.66

**Published:** 2023-04-03

**Authors:** Yixing Zhu, De Chang

**Affiliations:** ^1^ Graduate School of The PLA General Hospital Beijing China; ^2^ Department of Respiratory and Critical Care Medicine, Eighth Medical Center, Department of Respiratory and Critical Care Seventh Medical Center Chinese PLA General Hospital Beijing China

**Keywords:** adaptive immune response, chronic obstructive pulmonary disease, innate immune response, microbiome

## Abstract

Chronic obstructive pulmonary disease (COPD) is a common chronic respiratory disease and the third leading cause of death worldwide. Developments in next‐generation sequencing technology have improved microbiome analysis, which is increasingly recognized as an important component of disease management. Similar to the gut, the lung is a biosphere containing billions of microbial communities. The lung microbiome plays an important role in regulating and maintaining the host immune system. The microbiome composition, metabolites of microorganisms, and the interactions between the lung microbiome and the host immunity profoundly affect the occurrence, development, treatment, and prognosis of COPD. In this review, we drew comparisons between the lung microbiome of healthy individuals and that of patients with COPD. Furthermore, we summarize the intrinsic interactions between the host and the overall lung microbiome, focusing on the underlying mechanisms linking the microbiome to the host innate and adaptive immune response pathways. Finally, we discuss the possibility of using the microbiome as a biomarker to determine the stage and prognosis of COPD and the feasibility of developing a novel, safe, and effective therapeutic target.

## Highlights

- The lung microbiome of healthy individuals is compared to that of patients with chronic obstructive pulmonary disease (COPD).
- The role of the lung microbiome in the pathogenesis of COPD and host immune alterations is discussed.
- The most representative studies are presented for accuracy and comprehensiveness.

## Graphical abstract

The lungs possess a strong defense system of mucus, structural cells, immune cells, and extracellular matrix that clears or inactivates causative agents. The immune defense mechanism of the lungs consists of innate immunity and adaptive immunity, and mucociliary clearance is the first line of defense in innate immunity. Cells such as alveolar epithelial cells, macrophages, and dendritic cells bind to pathogen‐associated molecular patterns on their surfaces through pattern recognition receptors when a pathogen breaks through the immune defenses of airway epithelial surfaces and recognizes the corresponding pathogen. Various Tissue‐Resident Lymphocytes in the lungs secrete different cytokines stimulated by antigens, recruit effector cell subsets and provoke corresponding immune responses, eliminating pathogens. Adaptive immunity mainly includes cellular immunity mediated by T cells and humoral immunity mediated by B cells. The normal flora in the lungs also plays a good protective role for the host. They can also contend for ecological niches with opportunistic pathogens and foreign pathogens, decreasing the likelihood of pathogenic infection colonization.

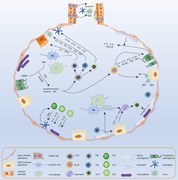

## 1 INTRODUCTION

Chronic obstructive pulmonary disease (COPD) is a common, preventable, and treatable chronic lung disease that poses a substantial burden on global health and the economy. According to the results of a 27‐year global cohort study published in *The Lancet*, the top three causes of death worldwide in 2017 were cardiovascular disease (31.8% of all death causes), cancer (17.1% of all death causes), and chronic respiratory disease (7.0% of all death causes). COPD is the most common cause of death from chronic respiratory diseases.^1^ As the third leading cause of death, COPD was responsible for 3.23 million deaths globally in 2019.^2^ Additionally, COPD is commonly associated with the presence of comorbidities, and as the global population ages, more people will die from COPD complications than from the disease itself.

Although the etiology and pathogenesis of COPD remain unknown, it is speculated that COPD results from the interaction between various environmental factors and host factors over time. Tobacco exposure is generally considered to be the primary environmental factor causing COPD.^3^ Other risk factors include occupational exposure to organic dust or chemicals; indoor air pollution from biomass fuel or coal combustion^4^; early life events that hinder lung development and maturation, such as intrauterine dysplasia, preterm birth, and lower respiratory tract infections^5^; infections caused by bacteria, fungi, viruses (such as SARS‐CoV‐2), mycoplasma, and other microbial infections^6^, ^7^, ^8^; and α‐1 antitrypsin deficiency.^9^ Microbial infection is an important factor in the pathogenesis of COPD. The lung was considered sterile for a long time owing to the limitations of culture techniques and the barrier function of the pharynx, which prevented isolation of the oropharynx and trachea. In 2010, Hilty et al. used 16S rRNA sequencing to detect microbiota in the lower respiratory tract of patients with asthma and COPD. Their findings confirmed the presence of a characteristic microbiota, challenging the traditional medical dogma regarding the sterility of the lower respiratory tract.^10^ Knowledge regarding the interactions between the lung microbiome and the host immunity is crucial to determine the role of the lung microbiome in the pathogenesis of COPD.

## 2 HEALTHY LUNG MICROBIOME

The microbiome refers to the entire microbial community (including bacteria, fungi, viruses, protozoa, and archea) as well as their genomes, metabolites, and interactions with the host's internal environment, which are present in a specific habitat at a specific time.^11^, ^12^ As the largest and most intimate window through which the body interacts with the external environment, the respiratory tract is exposed to the surrounding environment when the airway is open. A study measuring the internal surface area of the lungs found that the internal surface area of a normal lung with a lung volume of 3000 mL ranges from 29 to 40 m^2^, which is dozens of times greater than the surface area of the body.^13^ According to Hilty et al., at least 2000 bacterial genomes exist per square centimeter of the respiratory epithelium.^10^ A healthy lung harbors an abundant microbiome, which primarily comprises bacteria. Firmicutes, Bacteroidetes, Proteobacteria, and Actinobacteria are the dominant phyla, whereas *Pseudomonas, Streptococcus, Prevotella, Fusobacteria*, and *Veillonella* are dominant genera in the lung microbiome.^14^ Mycobiomes are also present in healthy lungs. An observational study revealed that *Candida, Malassezia*, and *Sarocladium* are the most common fungi in healthy lungs.^15^ However, another comparative study showed that pulmonary fungi are primarily composed of environmental agents, such as *Davidiellaceae, Cladosporium*, and some *Aspergillus* with low abundance.^16^ Additionally, a certain load of rhinovirus, coronavirus, and bocavirus can be detected in the lungs of healthy people.^17^

If the human body is compared to an ecosystem, different anatomical regions of the body are equivalent to different regions of the ecosystem, and each microbe in the human body is equivalent to a species in the ecosystem. In a healthy state, the microbiome composition in a specific area of the body is primarily determined by the migration and elimination of microorganisms, whereas in a diseased state, the local microenvironment and bacterial reproduction rate dominate the species richness. Microaspiration from the oral cavity is the main source of microorganisms in the lungs, and the composition of the microbiota in the lungs is in a dynamic balance between the entry and selective elimination of the transient microbiota.^18^ Factors such as anatomical structure, physiological distance, oropharyngeal microbiome load, cough reflex, mucociliary clearance, and immune response can affect the migration and elimination of microbiota, thereby altering the composition of the lung microbiome.^19^ When the host is sick, the internal environment of the lungs may be suitable for or inhibit the survival of certain microorganisms, causing the corresponding microorganisms to colonize, contract, or expand.

## 3 LUNG MICROBIOME IN COPD

Numerous studies have shown that the lung microbiome differs among healthy subjects, smokers, nonsmokers, and patients with stable and exacerbated COPD stages.^20^, ^21^, ^22^ The occurrence and development of COPD are closely related to the host microbiome. Compared to the lung microbiome of normal individuals, that of patients with COPD is composed of various potentially pathogenic microorganisms, including *Hemophilus* and *Moraxella*,^23^ in addition to the microbial species present in healthy subjects. Using culture‐dependent and culture‐independent methods, Einarsson et al. found that the diversity and distribution of the lung microbiome were considerably lower in patients with COPD than in healthy individuals. The relative abundances of Actinobacteria, Firmicutes, and Proteobacteria, which are the most common phyla in the lungs, did not vary significantly; nevertheless, a significant reduction was observed in the relative abundances of Bacteroidetes and some common anaerobic bacteria, such as *Prevotella, Veillonella*, and *Actinomyces*, in patients with COPD.^22^ Wang et al. analyzed sputum samples from healthy subjects and patients with different COPD states and found that the relative abundance of *Hemophilus* in the lungs of smokers and patients with COPD was greater than that in the lungs of nonsmokers and healthy subjects, and the relative abundances of *Moraxella, Streptococcus*, and *Actinomyces* in the lungs of patients with stable COPD significantly increased.^24^, ^25^ Sze et al. compared lung biopsy samples from patients with severe COPD (GOLD4 stage) and healthy controls and found that the proportions of Proteobacteria and Actinobacteria in the lungs of patients with COPD were slightly higher than those in the lungs of healthy individuals, whereas the proportions of Firmicutes and Bacteroidetes decreased.^26^ Some studies have reported that the major pulmonary fungi in patients with COPD are *Candida*, yeast, *Campylobacter*, and *Aspergillus*.^15^, ^27^ Adenoviruses, respiratory syncytial viruses (RSV), and multiple respiratory viruses can often be observed in the lungs of patients with stable COPD.^28^ Acute exacerbation of COPD (AECOPD) is closely linked to the microbiome. The relative abundance of proteobacteria in the lungs of patients in the exacerbation phase is higher than that in the stable phase.^29^ The relative abundances of *Hemophilus, Moraxella, Streptococcus pneumoniae*, and *Pseudomonas* increases, whereas the α diversity of their microbiomes decreases.^30^, ^31^ Patients with COPD having predominant proportions of *Aspergillus, Penicillium*, and *Curvularia* tend to have more frequent exacerbations and higher mortality and show a more severe systemic immune response to these fungi.^32^ Indoor and outdoor fungal sensitization factors can also contribute to frequent COPD exacerbations.^33^, ^34^ Additionally, colonization by *Pneumocystis jiroveci* also aggravates chronic small airway inflammation and airway remodeling.^35^ A systematic review involving 19 studies and 1728 patients demonstrated that AECOPD is primarily associated with the presence of rhinoviruses, RSV, and influenza viruses, all of which had a detection rate of more than 5%, whereas coronaviruses, parainfluenza viruses, adenoviruses, metapneumoviruses, and bocaviruses had a detection rate of 5%.^36^ The results of this study were consistent with those of several other studies.^34^, ^37^ Notably, coinfection with multiple viruses, such as rhinovirus, RSV, and adenovirus, tends to occur in patients with severe COPD.^38^ Overall, there is no remarkable difference in the types of lung microbiome in patients with COPD versus healthy people; however, the diversity and distribution of the microbial community are considerably decreased in patients with COPD and further decreased with the severity and exacerbation of COPD. The relative abundance of commensal bacteria decreases, the proportion of opportunistic pathogens increases, and some foreign pathogens appear. The results of different studies vary, possibly owing to the bias caused by specimen selection, detection method, and the source of patients with COPD. The major composition of lung microbes in patients with COPD is summarized in Table 1. Changes in the lung microbiome can also be used as a biological indicator for the prevention, diagnosis, and prognosis of COPD.

**Table 1:** Summary of studies utilizing either cultured or noncultured methods to describe the microbiome in COPD.

Year	Participants	Type of sample	Test method	Main findings	References
1994	Forty outpatients with stable COPD and 29 outpatients with exacerbated COPD	PSB	Aerobic and anaerobic cultures, bacterial identification, and susceptibility testing	The prevalence of *Haemophilus influenzae* and *Streptococcus pneumoniae* in stable COPD patients is high. Exacerbated COPD is related to bacterial infection.	[39]
2000	Twenty‐six received prednisolone first and placebo second. Thirty‐three received placebo first and prednisolone second	Induced sputum	Spectrofluorimetric assay	Eosinophilic airway inflammation causes airflow obstruction in some COPD patients, and corticosteroids work by inhibiting this mechanism.	[40]
2001	Eighty‐three patients with COPD	NS and blood samples	Detection by culture, PCR, and serology	Seventy‐seven viruses (39 [58.2%] rhinoviruses) were detected in 53 (64%) patients. Viral were associated with frequent COPD exacerbations, a longer symptom recovery period and higher plasma fibrinogen or serum IL‐6 levels.	[41]
2008	Seventy‐six AECOPD patients from two emergency departments	NS	Nested PCR	Respiratory viruses, such as RSV, can cause exacerbation in high‐risk COPD patients. Only 25% of AECOPD were related to viruses, including RSV (6); parainfluenza (3); influenza (3); rhinovirus (4); and human metapneumovirus (3).	[42]
2009	Two hundred and seven healthy individuals and 339 COPD patients (192 with inhaled corticosteroids and 147 without).	BALF	16S rRNA gene sequencing and RNA‐Seq technique	The relative abundance of Prevotella in COPD patients and healthy subjects was 33.5% and 47.7%, respectively, which was the most different genus in the lung microbiome between the two groups. A decrease in prevotella and an increase in moraxella correlated with the severity of COPD.	
2009	One hundred and eight AECOPD patients, 88% of them were admitted.	Nasopharyngeal aspirates (NPA), paired sera, and noninduced sputum	Polymerase chain reaction	One‐third of patients (31%) infected viral, 9% with influenza A, 7% RSV and 7% with PIV‐3. Bacterial pathogens were identified in the sputum of 49% of patients, most commonly *Staphylococcus aureus, P. aeruginosa*, and *H. influenzae*. Influenza and RSV are frequent contributors of AECOPD.	[43]
2012	Twenty‐one lung transplant subjects. Seven subjects were transplanted for COPD or emphysema, 7 for interstitial diseases, one for pulmonary hypertension, and six for suppurative lung diseases.	BALF and OW	Unbiased high‐density and fungal internal transcribed spacer sequencing	Compared with control subjects, the richness and diversity of lung microbiome were decreased in transplant subjects.	[44]
2012	Six former smokers with moderate COPD	Sputum, bronchial aspirate, BALF, and bronchial mucosa	PCR amplification and pyrosequencing of the 16S rRNA gene	The lung is not sterile in COPD patients and support the existence a different microbiome in different sites. Streptococcus, Prevotella, Moraxella, Haemophilus, Acinetobacter, Fusobacterium, and Neisseria being the most commonly genera identified.	[45]
2015	Adults with GOLD stage 4 COPD (*n* = 5) and healthy individuals (*n* = 4)	Samples of explanted lung	16S rRNA gene sequencing and host mRNA sequencing.	The host immune response to the lung microbiome is related to the pathogenesis of COPD. Microbiome diversity declined as emphysematous destruction increased. expansion of the Proteobacteria phylum was the most significant difference between healthy individuals and GOLD stage 4 COPD patients	[29]
2015	Twenty‐eight healthy subjects.	OW, NS, BALF, and gastric aspirate samples	16S rRNA gene sequencing	In healthy individuals, the microbiome of the lung overlapped those found in the mouth but were found at lower concentrations, with lower membership and a different community composition. And the nasal microbiome contributed little to the lung microbiome	[46]
2017	Subjects with moderate‐to‐severe COPD (*n* = 73) and severe asthmatic patients (*n* = 32)	Sputum mediators	Factor and cluster analyses, 16S rRNA gene sequencing	Revealed three subgroups of asthma and COPD exacerbations, Cluster 1 increased proportions of the bacterial phylum Proteobacteria. Cluster 2 increased proportions of the bacterial phylum Bacteroidetes. Cluster 3 increased proportions of the phyla Actinobacteria and Firmicutes.	[23]
2017	Adults with COPD (*n* = 18), smokers with no airway disease (*n* = 8) and healthy individuals (*n* = 11)	BALF	Molecular detection—Illumina MiSeq sequencing	Microbiome in the lower airways of patients with COPD is significantly different to that found in smokers and nonsmokers. There was more pseudomonas in the lower airway of patients with COPD, Bacteroidetes were more common in the control group. Community diversity (α and β) was significantly lower in COPD group than in healthy group.	[24]
2017	Thirteen healthy nonsmokers, 13 healthy smokers and 13 patients with COPD	Surgical lung tissue and lung Extracellular vesicles	16S ribosomal RNA gene sequencing	Extracellular vesicles consistently showed more operational taxonomic units (OTUs), higher Shannon index and a lower Simpson index than lung tissue.	[47]
2019	Subjects with COPD (*n* = 43) and healthy individuals (*n* = 16)	Sputum samples obtained by spontaneous expectoration or induced	16S ribosomal RNA gene‐based microbiome survey, host RNA microarray analysis, and proteomic assays	Haemophilus and Moraxella associated with COPD inflammation and exacerbations. Haemophilus was associated with stable and exacebated COPD, while Moraxella was associated with exacebated COPD.	[1, 22]
2019	Four hundred and fifty COPD patients (GOLD stages 2–4) and followed for a mean of 27 months.	NS and OW were taken during stable periods, at URTI onset, 10 days after the URTI and during an AECOPD	Multiplex nucleic acid amplification test.	The risk of exacerbation after URTI symptoms depends on the particular virus. The prevalence of stable COPD virus infection was low, and the top two viruses were rhinovirus (54.2%) and coronavirus (20.5%). These numbers were 35.7% and 25.9%, respectively, during AECOPD visits.	[48]
2020	Five hundred and ten COPD patients from UK sites of the BEAT‐COPD, COPDMAP, and AERIS cohorts.	One thousand seven hundred and six sputum samples were analyzed using COPDMAP and AERIS as a discovery data set and BEAT‐COPD as a validation data set	Sputum differential cell counts and 16S rRNA gene sequencing	The lung microbiome can stratify COPD into neutrophilic Haemophilus‐predominant, neutrophilic balanced microbiome, and eosinophilic subgroups.	[9]
2020	Healthy individuals (*n* = 47), stable COPD (*n* = 337), acute exacerbation of COPD (*n* = 66), and a longitudinal COPD cohort (*n* = 34).	Sputum and serum samples	DNA extraction, mycobiome sequencing, and specific‐IgE assays	The lung mycobiome in COPD is characterized by Aspergillus, Penicillium, and Curvularia, which associated with exacerbations and increased mortality, but not for antibiotics or corticosteroids.	[49]
2020	Two hundred severe COPD patients from Europe and North America and followed longitudinally for 3 years.	Sputum samples collected at stable, acute exacerbation and follow‐up visits.	Nucleic acid detection and 16 S ribosomal RNA gene sequencing	Geographic and longitudinal differences in the lung COPD microbiota were correlated with diverse outcomes. Moraxella and Haemophilus were 5‐fold and 1.6‐fold more likely to be increased during an exacerbation event. Human rhinovirus (13.1%), coronavirus (5.1%), and influenza virus (3.6%) were the most common virus in AECOPD.	[50]

Abbreviations: AECOPD, acute exacerbation of chronic obstructive pulmonary disease; BALF, bronchoalveolar lavage fluid; COPD, chronic obstructive pulmonary disease; IL, interleukin; NS, nasal swab; OW, oropharyngeal wash; PSB, protected specimen brush; URTI, upper respiratory tract infection.

## 4 ANTI‐INFECTION MECHANISM OF THE LUNG

Normal people inhale large amounts of air every day, and dust, poisons, and pathogens are high‐risk factors for various diseases. The lungs possess a strong defense system involving mucus, structural cells, immune cells, and extracellular matrix that clears or inactivates causative agents.^51^ Different pathogenic factors lead to different host immune responses. In this section, we focused on the anti‐infection mechanism of the lungs. The immune defense mechanisms of the lungs consist of innate and adaptive immunity, and mucociliary clearance is the first line of defense in innate immunity. When a pathogen enters the respiratory tract along with air, part of it is discharged from the body through expiratory movement. The other part is deposited on the surface of the respiratory tract or alveolar epithelium, binds to mucin in the mucus, and is transported to the pharynx with the movement of cilia and turbulence of the mucous layer before being coughed up or swallowed.^52^ The bulk of mucus is a network of water and a variety of mucins as well as a variety of functional proteins with antibacterial activity, such as secreted immunoglobulin A, lysozyme, lactoferrin, leukocyte protease inhibitor, and phospholipase A2.^53^ These antibacterial substances carry out preliminary removal or inactivation of pathogens. In addition, the body excretes foreign bodies and excess mucus through the cough and sneeze reflexes.^54^ Alveolar epithelial cells, macrophages, and dendritic cells bind to pathogen‐associated molecular patterns (PAMPs) on their surfaces through pattern recognition receptors (PRRs) when a pathogen breaks through the immune defenses of the airway epithelial surfaces and recognizes the corresponding pathogen. Currently, the main PRRs include the toll‐like receptor (TLR), C‐type lectin receptor (CLR), retinoic acid‐inducible gene I‐like receptor (RLR), and Nod‐like receptor (NLR).^55^ TLR‐2/4 is the most extensively studied PRRs targeting pathogens in the lungs. When different components of microorganisms combine with TLR, two signaling pathways, the MyD88‐dependent signaling pathway and the TRIF‐dependent signaling pathway, are primarily triggered. Various pro‐inflammatory cytokines and type I interferon are induced.^56^

Various tissue‐resident lymphocytes in the lungs secrete different cytokines stimulated by antigens, recruit effector cell subsets, and provoke corresponding immune responses, thereby eliminating pathogens.^57^ All three innate lymphoid cells (ILCs) were detected in the lungs.^45^ Group 1 ILCs principally consist of NK cells and noncytotoxic ILC1, which can secrete IFNγ. Group 2 ILCs mainly consist of ILC populations that generate TH2 cell‐associated cytokines (IL‑5 and IL‑13), whose maturation depends on GATA binding protein 3 and retinoic acid‐related orphan receptor‐α. Group 3 ILCs contain all ILCs that produce IL‐17 or IL‐22, and their maturation depends on the transcription factor retinoic acid‐related orphan receptors‐γt.^58^ Upon stimulation, these lymphocyte subsets generate signals that activate downstream effector cells and eliminate pathogens. For example, IL‐22 regulates IL‐2/IL‐2R22 production in the lungs and reduces the expression of SARS‐COV‐2 entry receptors, such as ACE2 and TMPRSS2.^59^

**Figure 1:**
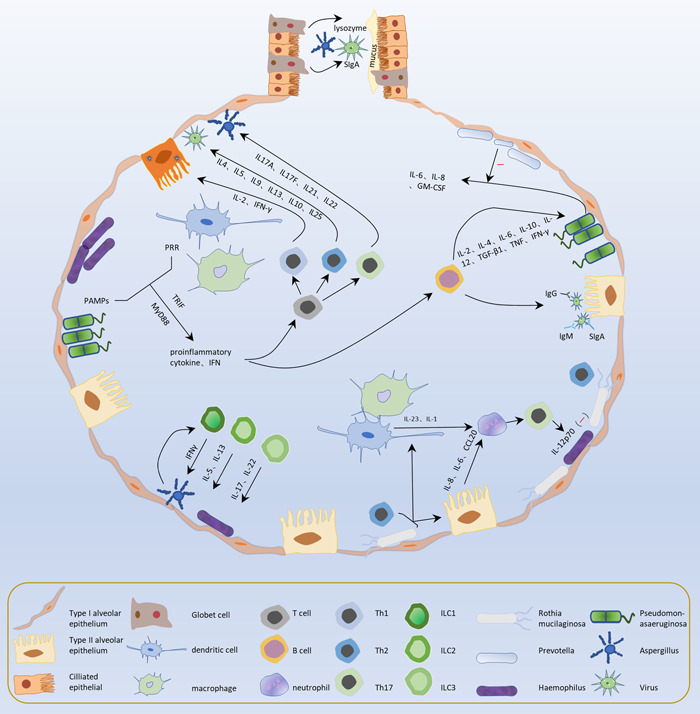
The lungs possess a strong defense system of mucus, structural cells, immune cells, and extracellular matrix that clears or inactivates causative agents. The immune defense mechanism of the lungs consists of innate immunity and adaptive immunity, and mucociliary clearance is the first line of defense in innate immunity. Cells such as alveolar epithelial cells, macrophages, and dendritic cells bind to PAMPs on their surfaces through PRRs when a pathogen breaks through the immune defenses of airway epithelial surfaces and recognize the corresponding pathogen. Various tissue‐resident lymphocytes in the lungs secrete different cytokines stimulated by antigens, recruit effector cell subsets and provoke corresponding immune responses, eliminating pathogens. Adaptive immunity mainly includes cellular immunity mediated by T cells and humoral immunity mediated by B cells. The normal flora in the lungs also plays a good protective role for the host. They can also contend for ecological niches with opportunistic pathogens and foreign pathogens, decreasing the likelihood of pathogenic infection colonization. GM‐CSF, granulocyte‐macrophage colony stimulating factor; IFN‐γ, interferon‐γ; IL, interleukin; IL‐12p70, Interleukin‐12p70; MyD88, myeloid differentiation factor 88; PAMP, pathogen‐associated molecular patterns; PRR, pattern recognition receptors; TNF, tumor necrosis factor; TRIF, Tir domain‐containing adaptor inducing interferon‐beta.

Adaptive immunity includes cellular immunity mediated by T cells and humoral immunity mediated by B cells. The innate immune system is essential for adaptive immunity. PRRs expressed by antigen‐presenting cells (especially dendritic cells) bind to PAMPs to activate the adaptive immune response,^60^ and cytokines secreted by other innate immune cells, such as interferon, can induce the proliferation and differentiation of T cells or B cells.^61^ CD8^+^ T cells are cytotoxic. Activated by antigen‐presenting cells, CD8^+^ T cells prolifically differentiate into cytotoxic effector T cells, which kill infected cells and release cytokines, such as granzyme B, IFN‐γ, IL‐12, IL‐2, and perforin.^62^ CD4^+^ Th cells can be divided into different subpopulations, including Th1, Th2, Th17, and regulatory T cells. After being stimulated by antigens, CD4^+^ Th cells rapidly differentiate and release related cytokines.^63^ Th1 cells mainly secrete inflammatory factors, such as IL‐2, IFN‐y‐, and TNF‐P, which mediate immune responses related to cytotoxicity and inflammation and activate other immune cells. Th2 cells secrete cytokines such as IL‐4, IL‐5, IL‐9, IL‐10, IL‐13, IL‐25, and amphiregulin to activate and maintain humoral immune responses against extracellular pathogens, allergens, and toxins.^64^ Th17 cells secrete cytokines such as IL‐17A, IL‐17F, IL‐21, and IL‐22, which mediate immune responses to bacteria and fungi.^47^, ^65^, ^66^ Follicular helper T cells can promote the maturation of germinal centers and regulate their functions by mediating protective immunity against pathogens.^67^ Regulatory T cells (Treg) suppress T cell immune responses, regulate immune tolerance, and prevent autoimmune diseases through cytokine (IL‐10, TGF‐β, and IL‐35) or contact‐dependent pathways.^68^ B lymphocytes secrete various antibodies, bind to surface proteins necessary for pathogens to enter cells to neutralize infection^69^ or bind to Fc receptors to exert antibody‐dependent cytotoxicity or activate complement responses.^70^ The most important antibodies in the lungs are dimeric SIgA and IgG, which are produced by airway epithelial cells.^71^ Furthermore, there are some multi‐reactive natural IgM autoantibodies that can not only resist the invasion of microorganisms but also regulate excessive inflammation caused by various factors to prevent autoimmune diseases.^72^ In addition to secreting antibodies, B lymphocytes can also present antigens and secrete cytokines including IL‐2, IL‐4, IL‐6, IL‐10, IL‐12, TGF‐β1, TNF, and IFN‐γ.^73^ It is worth mentioning that, like the symbiotic microbiota of the gut, the normal flora in the lungs also plays a protective role for the host (Figure 1).

## 5 PATHOGENESIS AND IMMUNOPATHOLOGY OF COPD

**Figure 2:**
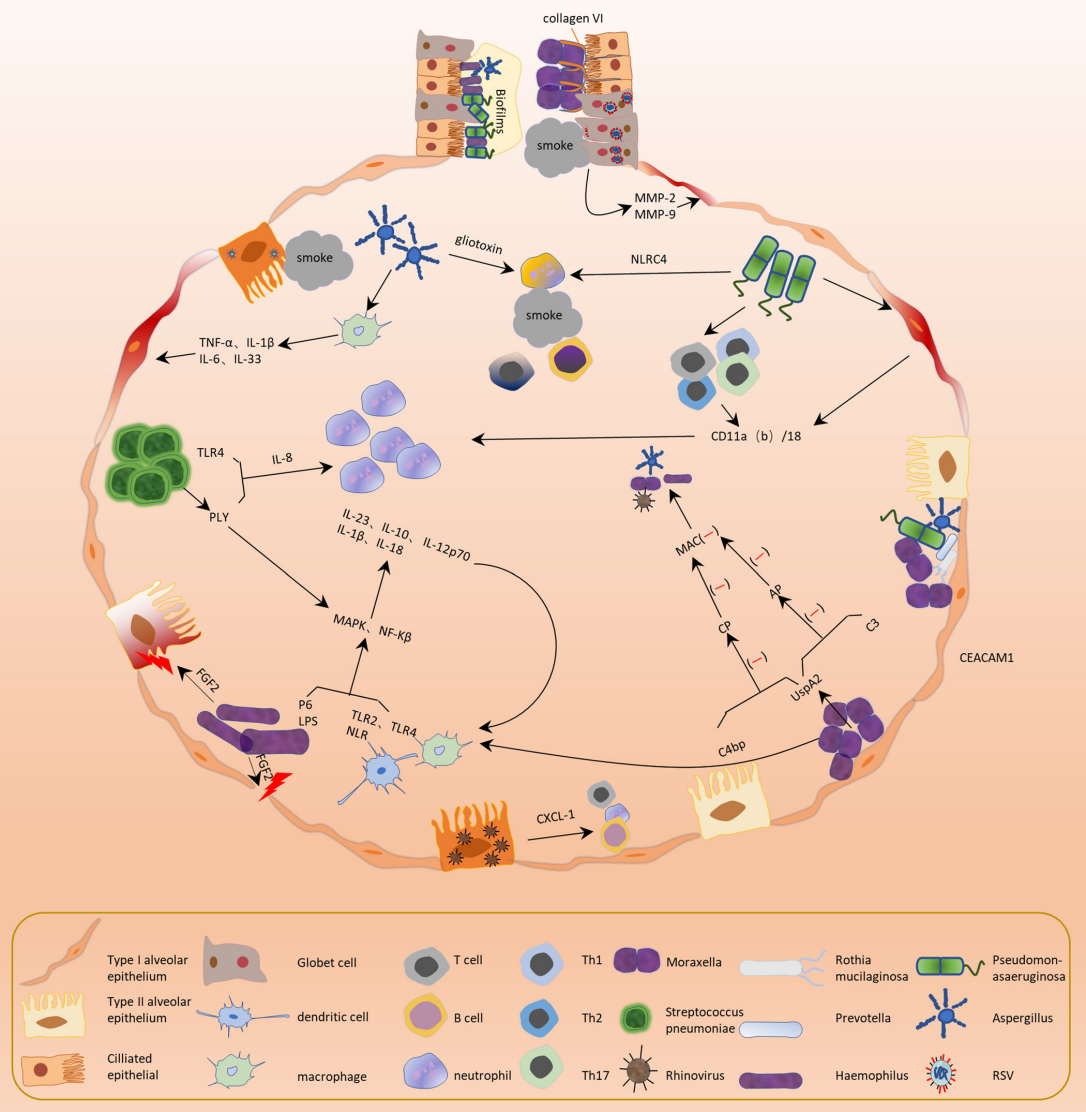
Changes in the microbiome interact with the host's immune response, leading to a “vicious cycle” in the process of chronic obstructive pulmonary disease (COPD), that is, multiple pathogenic factors damage the lung's defense mechanism, making the lung environment more suitable for pathogen infection or colonization. The opportunistic pathogens such as Haemophilus, Moraxella, Streptococcus, and Pseudomonas significantly increase. They cause airway remodeling and small airway inflammation by forming biofilms, releasing pathogenic factors, and inducing the production of numerous inflammatory factors. These alterations exacerbate inflammation in the small airway, which further deepens the damage to the lung's immune mechanism and expands the imbalance of the lung's microbiome. AP, alternative pathway; C4bp, C4b‐binding protein; CEACAM1, CEA cell adhesion molecule 1; CP, classical pathway; FGF2, fibroblast growth factor 2; IL, interleukin; LPS, lipopolysaccharide; MAC, membrane attack complex; MAPK, mitogen activated protein kinase; MMPs, matrix metalloproteinases; NF‐κB, (nuclear factor)‐κb; NLR, nod‐like receptor; P6, NTHI outer membrane protein 6; PLY, pneumolysin; TLR, toll‐like receptor; TNF, tumor necrosis factor; UspAs, Ubiquitous surface protein A molecules.

COPD develops as a result of a combination of multiple factors,^74^ chronic inflammation of the airway, pulmonary parenchyma, pulmonary blood vessels, imbalance of the protease‐antiprotease system,^75^ oxidative stress,^76^ autonomic dysfunction,^77^ nutritional disturbances,^78^ and other mechanisms that jointly cause small airway inflammation and emphysema changes. These pathological changes work together, resulting in COPD.^79^ Among them, the core is chronic inflammation of the small airways, in which the aggregation and activation of neutrophils, macrophages, T lymphocytes, and other inflammatory cells is a key link.^80^ According to different predisposing factors, pathogenesis, clinical phenotype, and severity, COPD can be divided into different types; however, there is no uniformly accepted classification. Factor and cluster analyses were used to analyze the cytokine profiles of patients, and COPD was divided into three inflammatory endotypes: neutrophilic inflammation, eosinophilic inflammation, and mixed cellular inflammation.^81^ Among these, neutrophil inflammation is the most common inflammatory endotype in COPD. When pathogenic factors contact the airway epithelium, the expression of various neutrophil chemokines (IL‐17, LTB, CXCL1, CXCL5, and CXCL8) increases, and neutrophils are recruited and activated in large numbers in the small airway, releasing neutrophil mediators such as neutrophil elastase and matrix metalloproteinases (MMPs), activating ILC3s, and mediating pathogen clearance. However, chronic excessive neutrophil inflammation and protease system imbalance can also lead to mucus hypersecretion, small airway damage, and airway remodeling.^20^, ^82^, ^83^ Neutrophils can also cause oxidative stress, which further aggravates airway inflammation. In addition, the formation of neutrophil extracellular traps (NETs) can be observed in the respiratory tract of patients with COPD in a positive association with the severity and frequency of progression.^84^ Once activated, neutrophils release a variety of proteases, peroxidases, and chromatin, which assemble outside the cell to form a fibrous trap to capture and kill microorganisms and release high concentrations of antimicrobial peptides to degrade bacterial virulence factors.^85^ The phagocytic function of neutrophils is in a state of competition with their ability to form Nets. When phagocytic function is impaired, neutrophils will turn to form many Nets to maximize the killing of pathogens.^86^ Macrophages are gatekeepers of the lungs. They can not only phagocytose foreign pathogenic factors and necrotic cell components in the host body, but also act as antigen‐presenting cells and secrete a large number of cytokines.^31^ Macrophages can be roughly divided into types M1 and M2, which mediate Th1‐dominated pro‐inflammatory responses and Th2‐related anti‐inflammatory responses, respectively.^87^ The number of M2 macrophages in the lungs of patients with COPD decreases as the number of M1 macrophages increases, indicating that the function of macrophages gradually transforms from anti‐inflammatory to pro‐inflammatory during the long‐term phagocytosis of foreign bodies.^88^ In addition, the ability of macrophages to phagocytose pathogens and necrotic cells is weakened in patients with COPD,^89^ leading to the immune escape of pathogens and the persistence of local inflammatory responses, further exacerbating the progression of COPD. The eosinophilic inflammatory endotype is also a rare type of COPD, but is associated with better corticosteroid prognosis,^90^ and Th2‐related immune microenvironments are more common in the lungs of these patients.^39^ Lymphocyte infiltration into the small airway wall increases with the progression of COPD, among which CD8 + T cells and B cells increase the most.^91^ Grumelli et al. found that emphysema‐related pathological changes in patients with COPD were strongly correlated with Th1‐related immunity,^43^ whereas the Th17 cell population and its related cytokines, IL‐17 and IL‐22, were strongly associated with the generation and exacerbation of COPD.^66^, ^92^, ^93^ On the one hand, IL‐17 and IL‐22 play a pivotal role in the clearance of pathogens.^94^ On the other hand, Th17 cells are closely associated with the development of small airway obstruction and emphysema. In mouse models of cigarette‐induced COPD, IL‐22 knockout mice had significantly lower lung function impairment than normal mice.^47^ This functional contradiction may be related to the different expression patterns of the two in time and space (Figure 2).

## 6 ROLE OF LUNG MICROBIOME IN THE PATHOGENESIS AND HOST IMMUNE ALTERATION IN COPD

Healthy lungs possess an extremely rich microbiome, and during the development of COPD, there is an imbalance in the original microbiome in the lungs and the invasion of foreign pathogens. These two conditions overlap to a certain extent, blurring the dividing line between colonization and infection. Changes in the microbiome interact with the host's immune response, leading to a vicious cycle in the process of COPD; that is, multiple pathogenic factors damage the lung's defense mechanism, making the lung environment more suitable for pathogen infection or colonization, exacerbating inflammation in the small airways, which further deepens the damage to the lung's immune mechanism and expands the imbalance of the lung microbiome. This can lead to the exacerbation of COPD.^95^ In the field of ecology, there exists the concept of core and hub microbiota, where “core microbiota” refers to the constant presence of certain microbiota in almost all related communities of a particular host. “Hub microbiota” refers to those microorganisms that have strong interactions with the host and other microorganisms, and even a minor change in their abundance is sufficient to alter the entire microenvironment.^96^ A similar core and hub microbiota is present in the lungs.^44^, ^97^ At the phylum level, the core microbiota of the lungs includes Bacteroidetes, Firmicutes, Proteobacteria, and Actinobacteria, and includes *Pseudomonas, Streptococcus, Prevotella, Fusobacterium, Hemophilus, Veillonella*, and *Porphyromonas* at the genus level. Numerous published studies have shown that the diversity of the lung microbiome is decreased, and the ratio of the core microbiota is broken in patients with COPD compared to healthy individuals. For example, the abundance of Bacteroidetes decreases while that of Proteobacteria increases, and the abundance of opportunistic pathogens such as *Hemophilus, Moraxella, Streptococcus*, and *Pseudomonas* significantly increases.^24^, ^30^, ^31^ At the same time, some foreign pathogens can also be observed in the lungs of some patients with COPD, such as *P. jiroveci* and SARS‐COV‐2.^8^, ^98^ Numerous functional redundancies are generated in the interaction between the microbiomes and the host, and thus the concept of functional microbiome has been introduced to classify microbiomes with analogous functions.^99^ Here, we focus on species that have a greater impact on the progression of COPD.

## 7 ALTERATIONS IN THE MICROBIOME AS A RESULT OF EXPOSURE TO RISK FACTORS

When the host is exposed to cigarette smoke, it attacks the peripheral immune system, resulting in damage to immune cells, making it difficult to maintain the effect of clearing infection. In addition, exposure to cigarette smoke may increase biofilm formation by specific bacteria, facilitating the reproduction and immune escape of pathogenic bacteria. Cigarette smoke also affects the microenvironment (e.g., oxygen, pH, and acids) in the lungs.^46^ The interaction of these mechanisms leads to changes in the composition of the microbiome in the lungs, with some anti‐inflammatory commensal flora being inhibited and pathogenic bacteria, such as *Pseudomonas aeruginosa*, expanding.^100^ The overuse of antibiotics also changes the diversity of the lung microbiome.^101^ For example, beta‐lactam antibiotic exposure is strongly associated with reduced microbial diversity in the lung,^102^ and macrolides can promote displacement of *Haemophilus influenzae* by *P. aeruginosa*.^103^

## 8 ANTI‐INFLAMMATORY COMMENSAL BACTERIA IN THE LUNGS OF PATIENTS WITH COPD

*Prevotella* belongs to the phylum Bacteroidetes and is an obligate anaerobic bacterium that is widely distributed throughout the human body. *Prevotella* has been observed to reduce IL‐12p70 production in *H. influenza*‐induced dendritic cells, thereby inhibiting Th1 cell‐associated pro‐inflammatory immunity.^104^ This may be caused by differences in the structures of the two lipopolysaccharides. Compared to the hexa‐acylated lipid A lipopolysaccharide of *H. influenzae*, the lipopolysaccharide of *Prevoella* is composed of penta‐acylated lipid A with lower activity. In the TLR4 signaling pathway, hypoacylated lipopolysaccharide binds to the small molecule glycoprotein MD‐2, competitively inhibiting hexa‐acylated lipopolysaccharides, thus reducing the inflammatory response caused by *H. influenzae* and others.^105^ *Prevotella* mainly binds to TLR2 to stimulate antigen‐presenting cells and airway epithelial cells to release IL‐23, IL‐1, IL‐8, IL‐6, CCL20, and other cytokines; recruit neutrophils; and activate Th17‐related immune responses, which are involved in establishing lung tolerance and improving respiratory tract anti‐infection ability.^49^, ^106^ In the lungs of patients with COPD, *Prevotella* competes with *Streptococcus, Hemophilus*, and *Moraxella*.^107^ The relative abundance of *Prevotella* decreases, and the inhibitory effect on opportunistic pathogenic bacteria is weakened, which also promotes the progression of COPD. *Rothia mucilaginosa*, a gram‐positive obligate anaerobe, performs a similar function. It not only inhibits the levels of IL‐6, IL‐8, GM‐CSF, and monocyte chemoattractant protein‐1 (MCP‐1) produced by pathogenic bacteria such as *P. aeruginosa* after invading lung epithelial cells, but also downregulates the expression of nuclear factor‐κB (NF‐κB) in epithelial cells through gene expression, dephosphorylation, and inactivation, thus suppressing inflammation of the small airways.^108^ Einarsson et al. found that the relative abundance of obligate anaerobes (*Prevotella, Veillonella*, and actinomycetes) was lower in the lungs of patients with COPD than in normal controls.^22^ These resident obligate anaerobes in the lungs participate in the production of short‐chain fatty acids (SCFAs), such as acetate, propionate, and butyrate, which can promote the recovery of airway barrier function, reduce the pathological changes associated with emphysema, and inhibit the proliferation of opportunistic pathogens.^78^ Commensal bacteria can also compete for ecological niches with opportunistic and foreign pathogens, decreasing the likelihood of pathogenic infection and colonization.^109^

## 9 POTENTIAL PATHOGENIC MICROORGANISMS IN THE LUNGS OF PATIENTS WITH COPD

The relative abundance of proteobacteria gradually increases in the lungs of patients with COPD at the stable and exacerbation stages, among which the most common species are *H. influenzae, Moraxella, S. pneumoniae*, and *P. aeruginosa*.^24^, ^25^, ^26^, ^110^ Interestingly, the bacterial load of *H. influenzae, Moraxella*, or *S. pneumoniae* in the lungs must reach a certain number to be able to cause COPD exacerbation, whereas low‐load *P. aeruginosa* can cause COPD exacerbation.^111^

*H. influenzae* is a facultative anaerobic gram‐negative bacillus, one of which, nontypeable *H. influenzae* (NTHI), is the most dominant pathogen in most studies of COPD microbiome.^112^ *H. influenzae* can also be observed in the lungs of healthy individuals, indicating that it is an opportunistic pathogen. When the respiratory tract is stimulated by cigarette smoke, biological contamination, or poisons, a variety of immunomodulatory pathways in the lungs are impaired, especially the ability to kill microorganisms, and *H. influenzae* changes from a colonizing to a pathogenic state, thus leading to the progression of COPD.^41^ Pettigrew et al. performed 15‐year whole‐genome sequencing of NTHI isolates from the lungs of patients with COPD. They found that NTHI alters its simple sequence repeats in the genome by slipped‐strand mispairing, thereby modulating its key functions in adapting to the changing lung environment.^113^ Four genetic islands, G2, G6, G8, and G10, are the most common in COPD strains, encoding potential transcriptional regulators and other small ORFG2, the entire urease operon, aspartate‐semialdehyde dehydrogenase, and ABC transporter, respectively. They regulate the formation of the corresponding products to facilitate the survival of NTHI in an acidic environment, maintain membrane integrity, and enhance drug resistance. In addition, it damages the respiratory epithelium via ammonia toxicity.^114^ For example, NTHI inhibits intracellular E‐cadherin production by inducing the expression of fibroblast growth factor 2, disrupting integrity and barrier functions in lung epithelial cells.^42^ NTHI can also express a variety of structural and functional proteins, enhance oxidative resistance and stress reactivity, increase nutrient uptake ability, improve adhesion activity, resist killing by antimicrobial peptides, and promote biofilm formation.^112^ Biofilms are how multiple microorganisms, including *H. influenzae*, colonize. It is composed of numerous extracellular polymeric substances and bacterial communities. Normally, the flow of gas and mucus in the lungs and the flapping of cilia cells limit biofilm formation.^115^ When COPD occurs, NTHI expresses a variety of attachment proteins, such as type IV pilus protein and adhesion protein OMP P1/P5, resulting in a large amount of mucous production, which impairs mucociliary clearance and biofilm form.^30^ Biofilms not only preserve bacteria for a long time, enhance their immune escape and antibiotic resistance, but also induce chronic inflammation in the host, further exacerbating the progression of COPD.^115^ NTHI can also express peroxiredoxin‐glutaredoxin and catalase to resist oxidative stress and escape killing by NETs.^116^ NTHI outer membrane protein 6 (P6) binds to TLR2 and activates p38 mitogen‐activated protein kinase (MAPK) or nuclear factor kappa B to induce the expression of endogenous anti‐inflammatory mediators such as cyclooxygenase 2 and prostaglandin E2.^117^ Hexa‐acylated lipid A lipopolysaccharides of NTHI bind to TLR4 and stimulate the production of numerous inflammatory cytokines. Jeppe et al. found that, owing to the high biological activity of hexa‐acylated lipopolysaccharide, cytokines such as IL‐23, IL‐12p70, and IL‐10 induced by pathogens such as *Hemophilus* and *Moraxella* in patients with COPD are 3–5 times that of commensal bacteria.^104^ NTHI can also bind to NLR, stimulate caspase‐1 expression, and secrete numerous IL‐1 family cytokines (IL‐1β and IL‐18), aggravating the pathological changes in COPD.^50^ All inflammatory mediators work together with NTHI to form positive feedback loops with the NF‐κB pathway as the core, which continuously enhances chronic airway inflammation and leads to COPD exacerbation.^41^

*Moraxella* is a facultative anaerobic gram‐negative bacillus belonging to the phylum Proteobacteria. Among the common pathogenic bacteria in the airways of patients with COPD, the colonization rate of *Moraxella* is second only to that of *Streptococcus* and *H. influenzae*.^107^ *Moraxella* binds to and adheres to collagen VI on airway epithelial surfaces. In COPD, the expression of this adhesion target is upregulated to recruit more *Moraxella* to colonize and adhere to the airway surfaces.^118^ Colonization is primarily associated with severe or exacerbated COPD. There are various functional proteins on the surface of *Moraxella*, the most important of which are ubiquitous surface protein A molecules (UspAs), which mediate the adhesion, invasion, nutrient acquisition, and immune escape of the bacteria.^48^ Among these, the ability to escape from complement immunity has the greatest influence on the pathogenesis of COPD. Complement is activated by the classical pathway, alternative pathway, or lectin pathway/mannose‐binding lectin, forming membrane‐attack complexes and killing microorganisms. *Moraxella* UspA2 inhibits the activation of classical and alternative pathways by binding to the complement inhibitor C4bp and absorbing serum C3, respectively, and decreases the activity of membrane attack complexes.^119^ In addition, *Moraxella* UspA1 binds to CEACAM1, an epithelial cell adhesion molecule, and inactivates the PI (3)K–NF‐κB signal transduction pathway through phosphorylation to escape from the clearance by the immune system.^120^ Like other opportunistic pathogens in the lungs, *Moraxella* stimulates airway epithelial cells to release inflammatory factors such as IL‐8 and GM‐CSF via MAPK and NF‐κB activation, and promotes the production of IL‐8 by acetylating histones H3 and H4.^121^

*S. pneumoniae*, an aerobic or facultative anaerobic gram‐positive coccus in the phylum Firmicutes, is another major opportunistic pathogen in the lungs after *Hemophilus*.^107^ Polysaccharide capsules and pneumolysins are the main pathogenic factors of *Streptococcus*. The negatively charged polysaccharide capsule generates electrostatic repulsion with mucopolysaccharides, helping *Streptococcus* escape mucous immune clearance and colonize the airway epithelial surface. The presence of a polysaccharide capsule can also help *S. pneumoniae* escape macrophage phagocytosis.^122^ Pneumolysins can interact with the host in various ways. First, it can activate the host complement system through the classical pathway. Second, it interacts with epithelial cells to form pores, causing “osmotic sensing” and activating downstream p38 MAPK and NF‐κB. Third, it binds to TLR4 and secretes chemokine IL‐8, recruiting neutrophils. Pneumolysin downregulates the expression of host antiproteases and combines with hydrogen peroxide generated by bacteria to induce apoptosis in host cells.^123^

*P. aeruginosa*, a gram‐negative obligate anaerobe in the phylum proteobacteria, can be isolated from patients with COPD during exacerbation or recurrent hospitalization.^124^ In the early colonization stage, the emergence of *P. aeruginosa* is always accompanied by lower cytotoxicity, motility, and protease expression levels and the formation of more biofilms, which is the mechanism of its long‐term colonization in the lungs.^125^ It exhibits extremely high variability and can vary in different microenvironments. In diverse environments, *P. aeruginosa* possesses LPS with different structures, such as penta‐acylated or hexa‐acylated lipid A, which leads to changes in its immunogenicity.^126^ *P. aeruginosa* releases quorum‐sensing signal molecules to promote mucus secretion and biofilm formation and inhibits the activity of immune cells, forming immune tolerance to the host.^127^ Neutrophils are the main defensive force against *P. aeruginosa* infections. On the one hand, *P. aeruginosa* stimulates airway epithelial cells and immune cells to synthesize chemokines such as CD11a(b)/18, which drives neutrophils to migrate to the lungs.^128^ On the other hand, it can activate NLRC4 inflammasome‐induced neutrophil apoptosis.^129^

Fungi also play a significant role in the development of COPD. In a healthy state, fungi in the lungs can regulate host immune function and protect the airway mucosa. When the host suffers from COPD, the composition of the pulmonary fungal community changes and the relative abundance of *Aspergillus* changes most dramatically.^130^ *Aspergillus* and other allergens are associated with poorer lung function and more severe GOLD stage, and cause elevated IgE levels in nearly half of patients with COPD.^131^ The most important risk factors for *Aspergillus* infection are low host immunity and neutropenia; neutrophils are the main forces of the host that kill *Aspergillus*.^132^ Neutrophils synthesize IL‐17A via dectin‐1‐ and IL‐23‐dependent pathways, providing a protective immune response to COPD complicated by invasive pulmonary aspergillosis.^133^ Furthermore, *Aspergillus fumigatus* induces neutrophil apoptosis by synthesizing secondary metabolites, such as gliotoxin, thereby evading clearance by the lung's immune response.^134^ When combined with TLR2/4 or non‐TLR PRRs dectin‐1 on the surface of pulmonary macrophages, *Aspergillus* upregulates the expression of high mobility group box 1, which stimulates macrophages to release TNF‐α, IL‐1β, IL‐6, IL‐33, and other inflammatory factors, leading to the exacerbation of COPD.^135^ In addition, the roles of other fungi, such as *P. jiroveci*, in the progression of COPD should not be neglected.^98^ *P. jirovecii* can secrete a variety of matrices either by itself or by inducing the airway. This disrupts the balance of the proteinase‐antiproteinase system in the body, resulting in aggravation of airway obstruction.^136^ It can also activate macrophages through an NF‐κB‐dependent pathway to release TNF‐α, IL‐6, IL‐8, and other cytokines. This pathway is mediated by the β‐glucan‐rich cell wall of *P. jirovecii* and occurs more slowly and persistently than the same response induced by bacterial LPS.^137^ The host upregulates the expression of INF‐γ, CXCL9, CXCL10, and CXCL11‐related genes and mediates the clearance of *P. jirovecii* through Th1‐related immunity, which also aggravates inflammation of the small airways.^138^

Exacerbation of COPD is closely related to respiratory viral infection. In a comparative study, half of the stable COPD cases are associated with bacteria (54.7%) or viruses (48.4%), compared with 17.2% and 42.2% in acute exacerbations, respectively.^139^ Rhinovirus is the most common virus in the lungs of patients with COPD. After entering the lungs, rhinovirus primarily invades airway epithelial cells and replicates in large quantities.^140^ Replication of viral RNA leads to durable expression of CXCL‐1, induces multiple immune cells to aggregate in the airways, binds to multiple PRRs in the host, activates transcription factors such as interferon regulators and NF‐κB, and then induces transcription of type I and III interferons and other cytokines.^141^ However, the activation of this antiviral immunity is delayed in patients with COPD compared to that in healthy individuals. Rhinovirus‐induced innate immunity peaks at 48 h in healthy subjects and is delayed to 96 h or even later in patients with COPD.^140^ Rhinovirus infection can also impair the innate immunity of the airway epithelium by inhibiting antimicrobial peptides and increasing oxidative stress in the lungs, further increasing the susceptibility of patients with COPD to pathogens.^142^ This result was also demonstrated in another study, in which Molyneaux et al. inoculated subjects with rhinoviruses in both patients with COPD and healthy individuals. Compared with the control group, the COPD group had a six‐fold increase in pulmonary bacterial load after 15 days, and a 16% increase in the proportion of Proteobacteria sequences dominated by *Hemophilus* that lasted up to 6 weeks.^143^ In a large prospective study involving 1099 adults hospitalized with RSV, patients with COPD were 3–13 times more likely to be infected than those without COPD.^144^ A few studies have shown that RSV infection can decrease type I IFN levels, increase IL‐17 and IL‐23 secretion, upregulate mucin gene expression (MUC5AC and GOB5), and destroy ciliated epithelial cells in COPD models, leading to mucus retention and alveolar cavity enlargement.^145^ In addition, RSV infection increases the synthesis of MMP‐2 and MMP‐9, facilitating small airway remodeling and alveolar wall destruction.^146^

In addition to bacteria, fungi, and viruses, atypical pathogens are associated with the occurrence of COPD. A randomized controlled trial in Korea found that approximately 5.6% of patients with COPD showed serological evidence of acute infection with *Mycoplasma pneumoniae*.^147^ Similar results were observed for *Chlamydia pneumoniae*, and macrophages released more cytokines, such as TNF‐α and IL‐8, after phagocytosis of *C. pneumoniae*.^148^ However, a Turkish study found no significant difference in *C. pneumoniae* infection between COPD and healthy individuals and no significant association between serological markers of *Chlamydia* infection and COPD severity.^149^ At present, there are few related studies, and some research results are quite different. The different results in these studies may result from the heterogeneity of COPD on the one hand, and the differences in experimental protocols, including specimen sources and detection methods, on the other hand. However, atypical pathogens may also induce COPD.

## 10 DISCUSSION

COPD is a chronic airway disease induced by numerous pathogenic factors that have been associated with multiple mechanisms. The role of microbiomes runs throughout the entire pathogenic process. In addition to the microorganisms themselves, the virulence factors released and downstream metabolites have an impact on COPD. After the combination of *P. aeruginosa* surface LPS with TLR4, the mitochondria of alveolar macrophages and neutrophils secrete massive succinic acid and reactive oxygen species, which cooperate with the activation of the inflammasome to induce the maturation of IL‐1β and its release into the extracellular space. *Staphylococcus aureus* can synthesize a low metabolic small colony variant in the lungs, which can affect the glycolysis process of the host by degrading fumarate, consequently affecting host immunity.^150^ As mentioned above, commensal microbiota in the lungs can strengthen host immunity and inhibit the proliferation of pathogens by synthesizing SCFAs. In addition, commensal microbiota provides numerous essential signals for the development and maturation of the host's innate and adaptive immune systems.^151^ There is a mutually beneficial cooperative relationship between the host and commensal microbiota, and a mutually restrictive hostile relationship between the host and pathogen. This relationship exists not only between the host and the microbiome but also within vast microbiomes. Commensal microbiota competes with pathogens for the niche through nutrient uptake and metabolite production, preventing the colonization and multiplication of pathogens. However, when the host is stimulated by endogenous or exogenous factors, immune function is impaired, the local microenvironment changes, and it is no longer suitable for the survival of commensal microbiota. Then, the pathogens expand sharply and occupy the living space of the commensal microbiota. Interestingly, the presence of closely related species increases the chances of a new species entering the ecosystem, which is known as “like will to like” in the study of the gut microbiome.^152^ A similar pattern was observed in the lungs. Wang et al. constructed a network of pulmonary microbiome interactions in patients with COPD exacerbations. They found that *Hemophilus, Moraxella*, and *Streptococcus* are exclusive, which is associated with reduced diversity of the lung microbiome during COPD exacerbations.^25^ Jacobs et al. found that, in stable COPD, *S. pneumoniae* and *H. influenzae* had an interspecific synergistic relationship, whereas *P. aeruginosa* showed an interspecific competitive relationship with *H. influenzae* and *Moraxella catarrhalis*. In the acute exacerbation of COPD, the interspecific cocolonization relationship between *S. pneumoniae* and *H. influenzae* disappeared, whereas *P. aeruginosa* continued to inhibit the reproduction of *H. influenzae* and *M. catarrhalis*.^153^ Similarly, the lungs have complex synergistic and antagonistic interactions with bacteria, fungi, viruses, and various atypical pathogens. Compared with infection alone, *H. influenzae* and rhinovirus coexposure synergize with the respiratory epithelium to stimulate CXCL8 and CCL20 production, leading to more severe airway inflammation.^154^ These complicated interactions are closely related to the structure of pathogens, metabolites, and host immunity, creating the possibility of multiple infections in patients with COPD and further exacerbating the progression of the disease. However, these interactions can also be used to guide the treatment of COPD; for example, the rational use of narrow‐spectrum antibiotics to specifically regulate the composition of the microbial community in the lungs, thereby indirectly affecting host immunity and improving the patient's condition.

The microbiomes in other parts of the host also influence COPD progression. The gut microbiome is closely related to the development of COPD, not only because the gut microbiomes can enter the lung through micro‐aspiration, but also because both the gut and lung originate from the endoderm and have similar anatomical structures, such as microvilli and cilia. The interaction between gut microbiomes and host immunity can not only enhance the immune function of the lungs and bring benefits to patients with COPD, but may also exacerbate the progression of the disease.^155^, ^156^ Intestinal microorganisms are major producers of SCFAs in the body. SCFAs generated in the intestine can be transported to the lung in various ways to enhance the barrier function of the respiratory epithelium and pulmonary immunity function, which plays a protective role in patients with COPD.^78^ One meta‐analysis showed that patients with *Helicobacter pylori* infection were 100% more likely to develop COPD than healthy people.^157^ In addition to gut microbiomes, oral microbiomes are associated with the occurrence of COPD. Recently, a few studies have shown that patients with stomatitis and periodontitis are more likely to develop COPD.^158^, ^159^ This may result from an increase in the number of pathogenic microorganisms in the unclean oral microenvironment, which increases the number of pathogen microaspirations into the lungs, ultimately leading to COPD.

During the development of COPD, the host immune system not only plays a role in resisting invasion, but also acts as a “destroyer.” When pathogens invade the respiratory tract of the host, innate and adaptive immunity in the lungs are successively activated. They then synthesize numerous cytokines, which cooperate with the commensal microbiota in the lungs to prevent colonization and eliminate pathogens. When subjected to various endogenous or exogenous stimuli, pathogens escape or tolerate the host immunity. The immune system is continuously activated. Epithelial cells, neutrophils, macrophages, and lymphocytes secrete large amounts of inflammatory mediators that act on normal lung cells and tissues, leading to small airway inflammation and alveolar wall damage. The relationship between pathogens and host immunity forms a vicious cycle that amplifies the cascade of airway inflammation and immune dysfunction.

## 11 CONCLUSION AND PERSPECTIVE

Owing to the development of next‐generation sequencing, microbiome studies are no longer limited to culture‐dependent methods. Research on the microbiome in the host has expanded from the gut to the whole body. Sequence analysis of conserved 16S RNA and 18S RNA sequences for bacteria and fungi is more precise and reliable than the results obtained by phenotypic identifications.^40^, ^160^ Subsequently, metagenomic analysis was used to further refine the identification results. These rapidly evolving technologies facilitate our understanding of the role of the microbiome in the host body and how they relate to each other. COPD is a chronic respiratory disease caused by a combination of mechanisms, in which the microbiome runs through the entire development process of the disease. The study of the COPD microbiome and host immunity can help us gain a deeper understanding of its pathogenesis and find targeted therapeutics. It can also guide existing treatment methods and contribute to further enriching the consensus regarding the diagnosis and treatment of COPD. However, most current studies on the COPD microbiome are at the cross‐sectional level, and owing to the heterogeneity of COPD, there are few long‐term prospective cohort studies with large sample sizes; therefore, it is difficult to determine the causal relationship between the microbiome and host immunity. At present, we prefer a two‐way causal relationship secondary to the trigger point, such as tobacco exposure. In addition, the human body is not solely spliced together by individual organs, but a complete microbiosphere. Networked connections exist between the microbiome and body in time and space. The environment in the body is in homeostasis, and endogenous or exogenous stimuli can disrupt this balance and cause a domino effect. Therefore, future studies on the interaction between the microbiome and host immunity in COPD should comprehensively measure the roles of other aspects, such as external stimuli (such as cigarette smoke, antibiotics, and glucocorticoids), microbiomes, and related metabolites in other parts of the body, as well as the interference of the autonomic nervous system.

## AUTHOR CONTRIBUTIONS

Yixing Zhu collected the literature and wrote this review. De Chang guided the revision of the structure and content of this review.

## CONFLICT OF INTEREST STATEMENT

The authors declare no conflict of interest.

## ETHICS STATEMENT

None.

## Data Availability

All data generated or analyzed during this study are included in this published article.
